# Are oligodendrocytes bystanders or drivers of Parkinson’s disease pathology?

**DOI:** 10.1371/journal.pbio.3002977

**Published:** 2025-01-08

**Authors:** José María Salazar Campos, Lena F. Burbulla, Sarah Jäkel

**Affiliations:** 1 Metabolic Biochemistry, Biomedical Center (BMC), Faculty of Medicine, LMU Munich, Munich, Germany; 2 Munich Cluster for Systems Neurology (SyNergy), Munich, Germany; 3 German Center for Neurodegenerative Diseases (DZNE), Munich, Germany; 4 Institute for Stroke and Dementia Research (ISD), University Hospital, LMU Munich, Munich, Germany

## Abstract

The major pathological feature of Parkinson ‘s disease (PD), the second most common neurodegenerative disease and most common movement disorder, is the predominant degeneration of dopaminergic neurons in the substantia nigra, a part of the midbrain. Despite decades of research, the molecular mechanisms of the origin of the disease remain unknown. While the disease was initially viewed as a purely neuronal disorder, results from single-cell transcriptomics have suggested that oligodendrocytes may play an important role in the early stages of Parkinson’s. Although these findings are of high relevance, particularly to the search for effective disease-modifying therapies, the actual functional role of oligodendrocytes in Parkinson’s disease remains highly speculative and requires a concerted scientific effort to be better understood. This Unsolved Mystery discusses the limited understanding of oligodendrocytes in PD, highlighting unresolved questions regarding functional changes in oligodendroglia, the role of myelin in nigral dopaminergic neurons, the impact of the toxic environment, and the aggregation of alpha-synuclein within oligodendrocytes.

## Introduction

**Fig 1:**
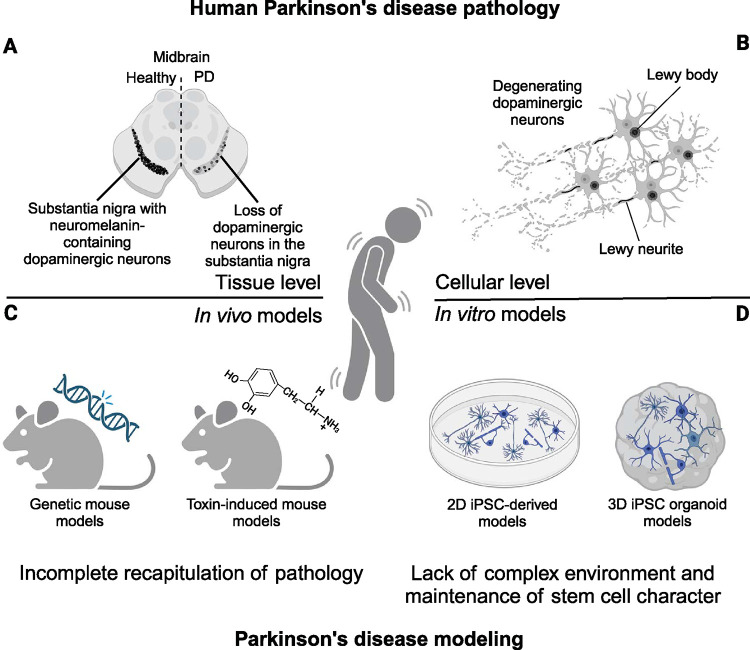
Disease modeling in PD. PD is a complex neurodegenerative human pathology that on the tissue level (A) is characterized by the loss of dopaminergic neurons in the substantia nigra in the human midbrain. On the cellular level (B), the major hallmark of PD pathology is the intracellular accumulation of α-synuclein into Lewy bodies and Lewy neurites in dopaminergic neurons and dopaminergic neuron degeneration. To model PD pathology, many genetic and toxin-induced mouse models (reviewed in [5]) have been developed (C). However, neither of those fully recapitulates human pathology, which is also likely due to species-specific differences between rodent and human biology [6]. Only a few models have specifically examined oligodendrocytes [7]. Therefore, human iPSC-derived 2D [8] and 3D models have been developed, including models with oligodendrocytes [9] with increasingly complex cellular compositions (D). Created with BioRender.com. iPSC, induced pluripotent stem cell; PD, Parkinson’s disease.

Parkinson’s disease (PD) is a progressive neurodegenerative disorder with a largely unknown etiology, with around 90% to 95% of cases being of sporadic origin. Estimated to affect 1% of the population above age 65 and 4% of the population above 85, it is the second most prevalent neurodegenerative disease and the most common movement disorder [1]. The two primary pathological hallmarks are the selective degeneration of dopaminergic neurons in the substantia nigra, and the accumulation and aggregation of the protein alpha-synuclein (α-synuclein) in Lewy bodies and Lewy neurites within neurons (Fig 1A and 1B) [1]. These features are integral to certain neurodegenerative processes, particularly in PD and Lewy body dementia. α-Synuclein is a small, soluble protein primarily found in the presynaptic terminals of neurons, where it is involved in regulating neurotransmitter release. In neurodegenerative diseases, this protein misfolds and aggregates, losing its normal function and becoming toxic to cells [2].

The neurodegeneration associated with α-synuclein, Lewy bodies, and Lewy neurites involves multiple mechanisms, i.e., pathological α-synuclein interfering with proper protein folding, mitochondrial function, neurotransmission, inflammation, and axonal transport of molecules and organelles. These processes collectively enhance neuronal vulnerability, particularly in regions like the substantia nigra, which results in the typical motor symptoms seen in PD [2]. Despite numerous advances in technologies and platforms, such as single nucleus-RNA sequencing [3], CRISPR gene editing, and induced pluripotent stem cell (iPSC)-derived disease modeling [4], the etiology of the disease remains elusive.

Initially, PD was viewed as a purely neuronal disease, which until recent years, directed research predominantly along a neurocentric path. However, with the rise of next-generation sequencing technologies, oligodendrocytes are increasingly coming into focus. Oligodendrocytes are highly abundant glial cells in the central nervous system, responsible for myelinating axons to facilitate fast saltatory nerve conduction and for providing support essential for neuronal function and long-term survival [10]. Oligodendrocytes have recently been suggested to play a significant role in the early stages of PD, as revealed by a strong link to PD risk genes and early transcriptional changes [11], which is further supported by evidence of structural changes in the white matter of patient brains [12,13]. As a significantly large volume of the white matter is comprised of myelin, these structural changes are believed to result, at least to a significant extent, from pathological alterations to oligodendrocytes. The functional relevance of these transcriptomic changes and their mechanistic effects are probably the most important open questions in the field at present.

Dopaminergic neurons in the substantia nigra have always been considered to be poorly myelinated [14]. Given the technological advances and the high abundance of oligodendrocytes in this area [15], this long-standing assumption may need to be re-evaluated, especially in the context of PD pathogenesis. Studying the non-myelinating functions of oligodendrocytes and their progenitor cells may also be of utmost importance in the future.

In this Unsolved Mystery, we provide a summary of the current, albeit limited, knowledge on oligodendrocytes in the pathogenesis of PD, discussing why they have remained such an enigma, and outline the necessary steps for future research to fully understand their functional implications. A better understanding of the non-cell-autonomous determinants of neuron vulnerability will be crucial for the development of novel approaches to disease-modifying therapies.

## Why has the role of oligodendrocytes in PD pathogenesis remained so elusive?

While postmortem brain tissue has revealed many insights into pathological aspects of PD, this approach is limited to a single snapshot of the late stage of the disease. The identification of pathological mechanisms heavily relies on suitable animal models in which the progress of disease can be studied in various experimental setups and in a time-dependent manner.

Rodent models for studying neurodegenerative diseases have become well established over the past 20 years, particularly genetic and toxin-induced models that recapitulate various aspects of the disease (reviewed in [5]) (Fig 1C). These models have indeed provided invaluable insights into the pathogenesis of PD, however with a strong focus on neurons. Animal models focusing on oligodendrocytes and their potential contribution to neuronal death and other pathological hallmarks of the disease are sparse. In a synthetic α-synuclein fibril-induced mouse model, one study reported the involvement of oligodendrocytes in α-synuclein processing and pathology progression in vivo, indicating that indeed oligodendrocytes may contribute to disease progression [7]. However, the inability of these models to fully recapitulate pathological hallmarks of the disease—especially in regard to oligodendrocytes—remains a major obstacle to the discovery of translational therapies.

Alternatively, in vitro models using iPSCs have been advanced to uncover the pathogenic mechanisms of PD (see Fig 1D and [4,8,16]), with a primary focus on neurons. However, ongoing advancements in these technologies offer exciting opportunities to explore both cell-autonomous and non-cell-autonomous effects of oligodendrocytes in PD (see Box 1).

**Box 1: Advantages and disadvantages of using stem cell-derived models for PD research**

Human iPSCs have gained increasing attention in scientific research since their discovery as this approach opened up an entirely new field of research for human disease modeling without the ethical concerns associated with the use of human embryonic stem cells. Not only does it allow modeling different aspects of the disease, including the generation of different cell types, but it has also paved the way for the development of more personalized medicine approaches, as data can theoretically be obtained from each individual patient.

Still, there are many critical views that iPSC-derived models retain a stem cell character even during differentiation and scarcely show signs of aging, which is a key feature of most neurodegenerative diseases, including PD. Unlike iPSC-derived neurons, oligodendrocytes can currently only be cultured in vitro for a very limited time before they begin to die, potentially making the problem of the lack of an aging signature in these cells even more pronounced.

Nevertheless, techniques such as direct cellular reprogramming offer an alternative way for producing lineage-specific terminal cells by transforming fully differentiated adult cells directly into a specific cell type, bypassing the pluripotent stage. Unlike the iPSC reprogramming technique, which resets the cell’s epigenetic information by reverting them to pluripotency, direct conversion focuses on introducing the epigenetic characteristics of the target cell. This allows the preservation of aging signatures [17,18] and their further development will greatly benefit the field of neurodegeneration. However, protocols to directly reprogram somatic cells into oligodendrocytes are yet to be established.

Protocols for the generation of iPSC-derived oligodendrocytes are still relatively rare in comparison to that of other cell types and difficult to establish, especially in co-culture systems with other cells. During development of the human brain, oligodendrocytes develop quite late and myelination only starts after birth, when most other cell types and structures have already been established [19]. Being made relatively late in the process of development is likely an explanation for why original protocols for generating iPSC-derived oligodendrocytes are lengthy [20,21]. Other, and significantly faster protocols that utilize overexpression of key oligodendroglial transcription factors [22,23] have already proven efficient, including in the context of patient-derived cell lines [9]. However, the generation of these genetically modified, or lentiviral-induced cell lines is laborious and difficult to reproduce due to variable virus integration site and transduction efficiency. Therefore, these protocols cannot be easily applied to different patient-derived cell lines that have not been modified. Oligodendrocytes are even more difficult to obtain in more complex 3D platforms, such as spheroids and organoids (Fig 1D). High numbers of oligodendrocytes are usually absent in these models, and although a few studies have shown active myelination in their model system [24,25], this key physiological property is generally lacking. Thus, generating more robust models that include physiological numbers of myelinating oligodendrocytes to fully represent their function in the human brain should be of highest priority in the future for oligodendrocyte-related research.

### Oligodendrocytes in Parkinson’s disease pathology: What do we know?

Magnetic resonance imaging (MRI) studies have shown that PD patients with dementia have significant structural changes in the majority of white matter tracts of the brain [12,13], often interpreted as demyelination. Although this type of measurement cannot directly assess the myelin content in patients, it may indicate cellular oligodendroglial changes or even a decline in the number of oligodendrocytes. Examining the peripheral nervous system, one study observed a significant loss of unmyelinated axons in the epicardial nerve of PD patients [26]. Even though this study does not strictly involve oligodendrocytes, as they only reside in the central nervous system, it highlights the neuroprotective function of myelin in the peripheral nervous system. In addition to the main pathological hallmarks, brain-wide oxidative stress is also an important feature of PD, and oligodendrocytes and oligodendrocyte progenitors cells (OPCs) are known to be highly vulnerable to oxidative damage [27]. This is due to a combination of high metabolic rates with potentially dangerous by-products, high intracellular iron, and low concentrations of antioxidants [28], further indicating that oligodendrocyte death may occur and contribute to the exacerbation of neuronal pathology.

Perhaps surprising for the field was the discovery of α-synuclein inclusions in oligodendrocytes in samples from PD patient brains [29,30]. This protein is thought to be expressed primarily in neurons, where it is known to regulate synaptic functions, and was therefore not expected to be present in oligodendrocytes. The source of α-synuclein remains under debate, as single nucleus RNA-sequencing approaches revealed endogenous expression of *SNCA*, the gene that encodes for α-synuclein, in oligodendrocytes in the human brain [31]. Oligodendrocytes from a rodent immortalized cell line are also capable of taking up monomeric and oligomeric forms of this protein in vitro [32]. Although this phenomenon is not very well defined in the context of PD, oligodendroglial α-synuclein inclusions are the main hallmark of multiple system atrophy, a rare neurodegenerative disease which also presents with parkinsonism [33]. Given that these diseases share some clinical and histopathological features, the question naturally arises as to how oligodendrocytes may be affected in PD and how they, in turn, respond to the pathological progression of the disease.

Imaging- and histology-based techniques for assessing oligodendrocytes in the context of PD are sparse, and interpretations of how these observations relate to the pathological developments are difficult. Due to technological advances in next-generation sequencing, studying oligodendroglial transcriptomic signatures in human patients has become easier. Indeed, recent single-cell and single-nuclei transcriptomic studies on human samples of the whole midbrain, the substantia nigra and other associated structures [11,15,34–39], have identified that oligodendrocytes are, after neurons, likely the second most affected cell type in PD (a detailed summary of study results is given in Table 1). Although these datasets largely vary in size, ranging from 17,000 to 316,000 analyzed nuclei, common findings were that oligodendrocytes always comprise the largest cell population in these brain regions and that the transcriptional landscape of oligodendrocytes was always altered. Depending on the detail and focus of the analysis, oligodendrocyte numbers are significantly depleted [39], or shifted in their proportional composition of the subpopulations, with some being depleted, but some also enriched [38]. The identified transcriptional alterations that were identified in PD oligodendrocytes were largely variable across studies, including an increase in stress response [34], inflammation [36], or response to unfolded protein [39] alongside an apparent loss of their myelinating capacity [36,39]. The differences in these findings affecting functional changes could likely be derived from different samples sizes, with largely variable gene numbers and different bioinformatic methods being applied. Most of these studies have combined these data with genome-wide association studies (GWAS) [9,11,35] or other means to include genetic risk-loci [15] and found that the genetic risk in PD is associated not only with dopaminergic neurons, as expected, but also with oligodendrocytes, and that oligodendrocytes can also be associated with clinical outcome prediction [37]. Furthermore, simultaneous use of single-nucleus RNA- and ATAC-sequencing of the human substantia nigra has also revealed that differential changes present in oligodendrocyte transcriptomes can be particularly associated to pathology progression [34].

**Table 1:** Summary of the major human single-cell transcriptomic studies applied on postmortem brain tissue in the context of PD.

Publication	Experimental design and brain region	Major findings
**[34]**	snRNA-seq (58,000 nuclei)/snATAC-seq in young, aged, and diseased substantia nigra nuclei. Data derived from sporadic PD patients.	Oligodendrocytes undergo transcriptional changes during aging and even further in PD, with little change in chromatin accessibility. Disease-associated oligodendrocytes are characterized by loss of a myelination signature and genes associated with neuroprotection, as well as an increase in stress response.
**[35]**	snRNA-seq (17,000 nuclei) in cortex and substantia nigra in combination with publicly available GWAS data. Data derived from sporadic PD patients.	The substantia nigra has much higher proportions of oligodendrocytes than the cortex, and oligodendrocytes are, alongside with neurons, associated with PD risk loci, involving pathways that are (among others) associated with metabolic processes and gene regulation.
**[36]**	snRNA-seq (19,000 nuclei) in putamen. Data derived from sporadic PD patients.	PD-associated oligodendrocyte populations exhibit increased inflammation, misfolded proteins, and reduced myelination capacity, which can be recapitulated in an animal model of α-synuclein pathology.
**[11]**	Large-scale reanalysis of publicly available bulk-RNA-seq and snRNA-seq data from mouse and human central nervous system in combination with GWAS data.	Oligodendrocytes are, alongside neurons, highly associated with PD risk loci. Oligodendrocytes in PD mostly react with up regulation of genes, suggesting increased activity or proliferation. These transcriptional changes happen in early Braak stages prior to onset of neurodegeneration.
**[37]**	snRNA-seq (89,000 nuclei) in prefrontal cortex and anterior cingulate regions in combination with publicly available GWAS data. Data derived from familial PD patients (LRRK2 and GBA1 mutations).	Oligodendrocytes are associated with PD risk loci, and specific subclusters can be linked to clinical outcome prediction for PD patients such as cognitive assessment and depression. Dysregulated PD-associated gene pathways in oligodendrocytes include regulation of tau-protein kinase activity, regulation of inclusion body assembly and protein processing involved in protein targeting to mitochondria.
**[38]**	snRNA-seq (84,000 nuclei) and spatial transcriptomics in human substantia nigra. Data derived from sporadic PD patients.	Oligodendrocytes are the largest cell population in the substantia nigra and their subpopulations show a shift in proportions in comparison to the controls, with 2 subpopulations majorly depleted, of which one expresses tyrosine hydroxylase and the other one is associated with iron storage and stress response. Overrepresented clusters are associated with pathways such as RNA polymerase complex function, lysosomal activity, MAP kinase activity, and microtubule organization.
**[39]**	snRNA-seq (41,000 nuclei) in the midbrain. Data derived from sporadic PD patients.	Oligodendrocyte numbers are significantly decreased in PD, as confirmed by immunohistochemical analysis. The loss of oligodendrocytes is linked to deficits in myelination, while the remaining oligodendrocytes appear to be involved in the cellular response to unfolded proteins.
**[15]**	snRNA-seq (316,000 nuclei) in the substantia nigra. Data derived from sporadic PD patients.	The PD-associated gene LRRK2 was strongly associated with oligodendrocytes in healthy samples and SNCA was strongly upregulated in diseased oligodendrocytes.

GWAS, genome-wide association studies; PD, Parkinson’s disease; snATAC-seq, single nucleus ATAC-sequencing; snRNA-seq, single nucleus RNA-sequencing.

Particularly surprising in this context is the apparent down regulation of genes involved in myelination, possibly indicating that this particular cell type simply becomes dysfunctional, or that oligodendrocytes need to take on other, as yet unknown, functions [34,36]. This finding is puzzling because dopaminergic neurons in the substantia nigra, the primary cell type affected in PD, are considered to be sparsely myelinated [14], at least according to common knowledge (discussed in the next section). In addition, a stress responding and inflammatory transcriptomic profile has been brought to attention in oligodendrocytes from samples of the striatum and the entire midbrain [34,35]. These results rather suggest a complex functional influence of oligodendrocytes in the pathogenesis of PD that—if oligodendrocytes become affected—goes beyond simple myelin loss and potential cell demise, although clear conclusions are difficult to draw, as human single-cell transcriptomic data come from a single time point of pathology.

In summary, the limited studies on oligodendrocytes in PD provide first evidence of non-cell-autonomous changes associated with reduced functionality of oligodendrocytes that may have widespread effects on the susceptibility of dopaminergic neurons, and therefore raise the hypothesis that these changes may potentially play a role in contributing to disease pathogenesis. While valuable, these studies are largely descriptive and indicate a change in the transcriptional profile of oligodendrocytes. Thus, further studies are needed to shed light on the functional impact of these changes on oligodendrocyte dynamics, and potentially reveal underlying molecular mechanisms contributing to pathology.

## Oligodendrocytes in Parkinson’s disease pathology: What remains unknown?

### How severely are oligodendrocytes functionally altered in Parkinson’s disease?

Given the discoveries of transcriptional changes in oligodendrocytes in PD highlighted above, several important questions for the field need to be raised. First, do oligodendrocytes play an active role in the pathogenesis of the disease or are they merely secondary victims of the pathological changes in affected neurons? Second, do these cells initiate and promote disease progression in early stages, or do they only respond to global damage from neighboring cells? Literature indeed suggests the possibility of an early active pathogenic role of oligodendrocytes. For example, the transcriptomic studies from human samples reported a shift in their gene expression profile as early as Braak stages 1 and 2 [11]. Nevertheless, these data only represent a single time point in the course of the disease. It is therefore impossible to say what happens in earlier stages of the disease and whether these transcriptomic changes are preceded by other physiological changes. We believe that appropriate human in vitro models could aid address these pathologies and, in long term, could lead to the development of new research strategies and potential therapies in the future.

The ability to combine single-cell transcriptomic and GWAS has enabled researchers to better understand which cell types carry disease-associated single-nucleotide polymorphisms (SNPs) and has contributed tremendously to bringing oligodendrocytes into focus [11]. However, how these SNPs influence the function of oligodendrocytes and in turn disease progression remains to be elucidated. As demonstrated in one study [34], the multi-omics approach that combines single-cell RNA-sequencing with single-cell ATAC-sequenzing (to assess genome-wide chromatin accessibility) on the very same cell, offers the possibility—albeit limited—of understanding which genes are affected by the SNP in a particular cell type. Further development of these combined technologies will greatly improve our understanding of the cell-specific pathological mechanisms in PD. Identifying and understanding these mechanisms requires an enormous collaborative scientific effort, and longitudinal studies using animal models and human patient material would be of great value to the field. This may also answer the question of whether oligodendroglial changes progress linearly or whether the observed pathological alterations also depend on different disease stages. This could open a whole new set of biomarkers that would aid patient care and prognosis.

### Does demyelination in the substantia nigra play a role in Parkinson’s disease?

**Fig 2:**
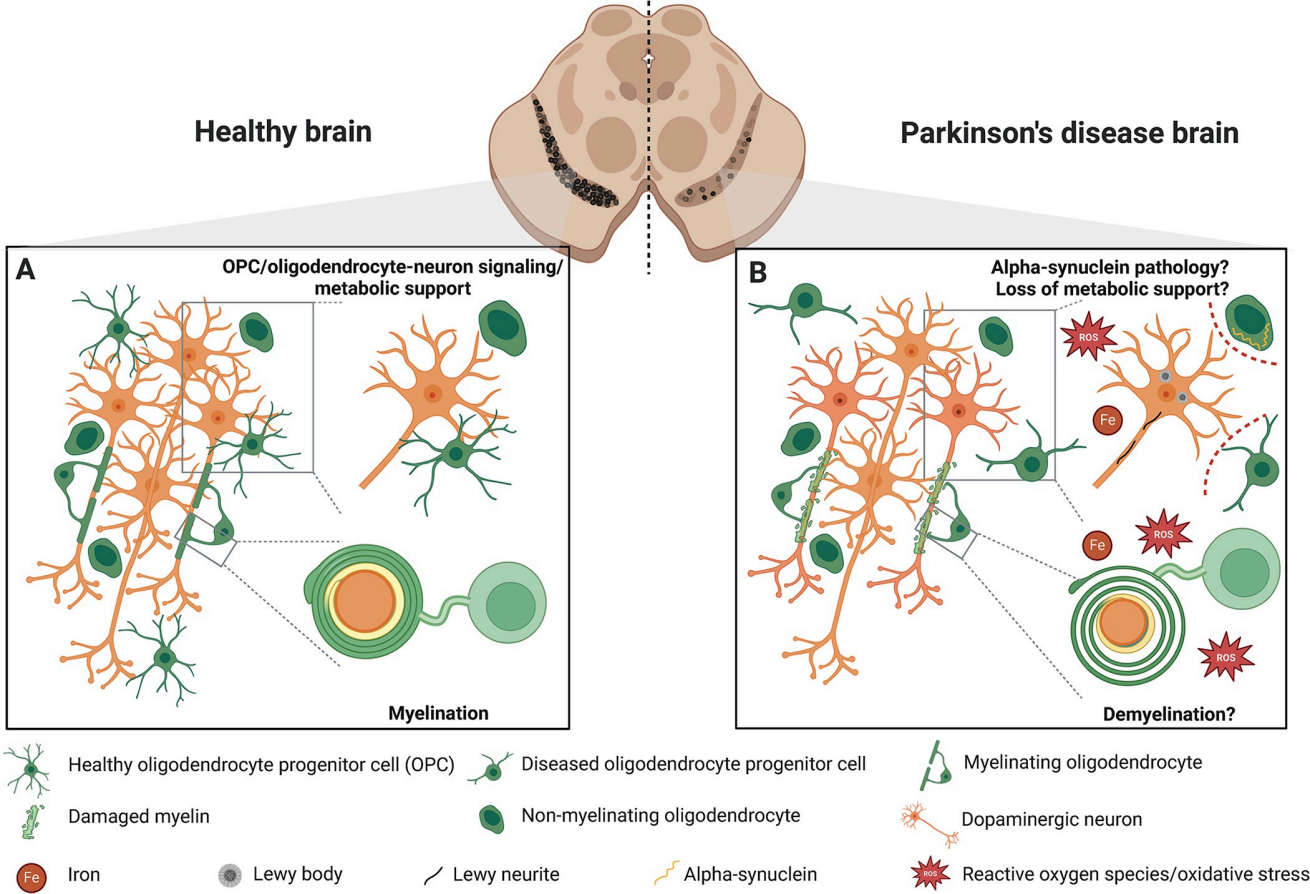
Function and dysfunction of oligodendrocyte lineage cells in healthy and PD brains. (A) In the healthy brain, the primary role of oligodendrocytes is the myelination of axons, though dopaminergic neurons are generally thought to have minimal myelination [14]. Moreover, both OPCs and non-myelinating oligodendrocytes are recognized for their crucial roles in signaling and providing metabolic support [10], although the exact function of the non-myelinating oligodendrocytes is even less clear. (B) In the PD brain, the particular consequences of damaged or non-functional oligodendrocytes is not well understood. Although there are reports of demyelination in patient brains [12,13], it is still unclear to what extent this affects dopaminergic neuron health or whether demyelination precedes neuronal damage. α-synuclein positive inclusions have been found in oligodendrocytes in PD patient brains [29,30]; however, it remains unclear whether this is due to cell-autonomous build-up of α-synuclein or protein cell-to-cell transfer from neurons to oligodendrocytes. It is hypothesized that diseased oligodendrocytes and OPCs may lose their direct signaling and metabolic support functions (indicated by dashed red lines) to neurons due to pathological changes in their environment, such as the accumulation of ROS and iron (Fe) accumulation that could exacerbate neuronal damage. Created with BioRender.com. OPC, oligodendrocyte progenitor cell; PD, Parkinson’s disease; ROS, reactive oxygen species.

Myelination in patients with PD has been largely assessed by MRI, which, as previously mentioned [12,13], is only an indirect measurement of myelin content; therefore, the question of whether demyelination is a common feature in PD remains largely speculative (Fig 2). Therefore, a thorough examination of the myelin content in patients at different stages of the disease will help us understand whether myelin changes precede and therefore could be causative of neuronal loss, or whether sublethal damaged neurons cause oligodendroglial changes. The general assumption in this field of research is that the degenerating dopaminergic neurons in the substantia nigra are only sparsely myelinated [14], which is probably one reason why the study of oligodendrocytes and myelin has not been the focus of research to date. However, it seems that the low degree of myelination of human dopaminergic neurons has fallen into a state of common knowledge, with actually little experimental evidence to support this statement.

On the contrary, newer studies, showing, for example, high proportions of oligodendrocytes [15,34,35,38] (summarized in Table 2) and high levels of myelination in the substantia nigra [40], suggest a higher degree of myelination of dopaminergic neurons than previously assumed. Additionally, improved technologies have shown that many neurons in regions of gray matter are only intermittently myelinated [41], demonstrating that even small fragments of myelin along axons are critical for complex neuronal functions [42]. Given this updated information, the myelin content of dopaminergic neurons should be reassessed using state-of-the-art technologies. Furthermore, it needs to be considered that PD is characterized by more widespread pathology in other brain regions and involves non-dopaminergic neurons as well. Hence, demyelination and malfunction of other neuronal populations may also contribute to motor and non-motor symptoms associated with disease progression. Finally, the high number of oligodendrocytes in areas with little apparent myelination suggests that myelination is likely not the sole purpose of these cells, and that their contribution to normal brain physiology may be far more pleiotropic than previously thought.

**Table 2:** Summary of the current state of the literature on the myelination state of the human substantia nigra.

Evidence for a sparse myelination in the substantia nigra		Evidence for a higher degree of myelination in the substantia nigra	
Publication	Information provided	Publication	Information provided
**[43]; original research article**	Heavily myelinated areas are less immunoreactive to α-synuclein staining, less myelinated areas are strongly immunoreactive to labeling with α-synuclein including the substantia nigra	**[34]; original research article**	Oligodendrocytes are the most prominent cell type captured from human substantia nigra shown through snRNA-seq and snATAC-seq
**[14]; commentary**	Proposes that neurons with heavily myelinated axons resist PD-related pathology, since myelinated neurons are more resistant to pathology, while poorly myelinated neurons are more vulnerable to pathology	**[35]; original research article**	snRNA-seq of human substantia nigra shows a higher proportion of nuclei from glia in the SN represented mainly by oligodendrocytes than that obtained from the cortex
**[44]; review including some original research data**	Description of the anatomy and functions of the limbic system. Discusses predominance of thin, unmyelinated fibres of the overall system and shows examples of the periaqueductal gray, a region close to the substantia nigra	**[38]; original research article**	Nearly 50% of all captured nuclei by snRNA-seq of human substantia nigra were from oligodendrocytes or OPCs
		**[40]; original research article**	Electron microscopy shows nearly 40% myelinated axons in the human substantia nigra
		**[15]; original research article**	Oligodendrocytes are the most prominent cell type captured from human substantia nigra shown through snRNA-seq

### Do oligodendrocyte functions deteriorate due to oxidative stress?

There is strong evidence for an imbalance of brain iron in PD associated with pathology, and the presence of elevated oxidative stress levels is an established pathological feature [45]. Oligodendroglial iron has not yet received much scientific attention in the context of PD, despite oligodendrocytes having the highest iron content in the central nervous system [46], essential for their metabolism. Given the complicated and intimate nature of communication between neurons and oligodendrocytes (Fig 2) and their antioxidant function related to iron metabolism [47], we raise the question whether oligodendrocytes exacerbate or mitigate neuronal iron overload and its associated neurotoxicity. Oligodendrocytes have been shown to be particularly vulnerable to oxidative damage [48], but there is still little known about the sources of reactive oxygen and nitrogen species and the effects on specific functions of oligodendrocytes, particularly with regard to direct coupling of neurons. We speculate that these toxic agents in the environment lead to impaired oligodendrocyte metabolism, rendering them incapable of providing their physiological protection to neurons, or, alternatively, oligodendrocytes themselves could be the source of these toxic components. Therefore, it should be considered that antioxidant support from oligodendrocytes on neurons [47] may still be present, but disease conditions overwhelm the process as the disease progresses.

### What is known about the spread of α-synuclein pathology between oligodendrocytes and neurons?

Evidence of oligodendroglial α-synuclein aggregates in PD has recently emerged [29,30] (Fig 2), which was initially thought to be due to oligodendroglial uptake of monomeric and oligomeric forms of α-synuclein [32]. However, more sensitive approaches have revealed that oligodendrocytes do express α-synuclein mRNA and protein [49]; yet the functional and disease-associated implications of this finding remains to be studied. To this end, we propose that advanced disease modeling platforms may be beneficial, such as co-culture systems of neurons with oligodendrocytes, to help assess whether oligodendrocytes are capable of taking up neuronal α-synuclein, thereby reducing the pathological burden, and how this may affect progression of pathology. Or, as another possibility, whether an abnormal function or level of α-synuclein in oligodendrocytes may account as a triggering determinant of pathology affecting α-synuclein levels and accumulation in neighboring neurons. In-depth investigation of this topic is critical to better understand possible risk factors and treatment strategies in PD, as well as other synucleinopathies.

### What role might oligodendrocyte progenitor cells play in the pathogenesis of Parkinson’s disease?

Since OPCs were considered merely precursors of oligodendrocytes for a long time after their discovery, many non-canonical functions of OPCs have been discovered, which have been extensively discussed previously [50]. Although not much is known about the contribution of OPCs to PD pathology yet, many of their functions could be of potential importance in disease pathogenesis (Fig 2). Several publications have shown that OPCs are important for neuronal function and their deficiency induces depression-like behavior [51] or obesity [52] in mice. Due to their strong synaptic coupling with dopaminergic neurons [53] (Fig 2), malfunctioning OPCs could disrupt cellular crosstalk, which could, in the long term, lead to functional impairment of this vulnerable cell type. Moreover, OPCs are capable of synapse pruning [54], which may be of pathological significance given the severe synapse loss in PD [55]. Lastly, OPCs have also been shown to have immunomodulatory functions via direct cytokine signaling [56] or through the acquisition of properties of antigen-presenting cells in multiple sclerosis [57,58], a chronic inflammatory condition resulting in oligodendrocyte death and demyelination. With regard to PD, an immunomodulatory function of OPCs could potentially exacerbate neuroinflammation.

### Oligodendrocytes in Parkinson’s disease pathology: What are the major challenges?

In recent years, studies using human brain tissue and next-generation transcriptomic approaches have proven indispensable for describing previously unknown pathological changes. However, the tissue is a limited resource and possible artefacts (due to non-standardized handling protocols of the postmortem tissue) is a source of great variability in the data. Furthermore, human brain tissue only provides a snapshot of the disease, and cellular and molecular changes that occurred long before disease onset remain speculative, making additional approaches essential.

Based on the Alzforum database (www.alzforum.org), there are currently 33 genetic and several other drug-induced rodent models of PD available. Although they span over a variety of risk genes, pathological features, and several molecular mechanisms, they all aim at reproducing well-known key pathological hallmarks of the disease and may therefore have limited relevance to oligodendrocyte biology. Moreover, mouse oligodendroglia differ significantly from human oligodendroglia at a fine transcriptomic level [59], which could potentially result in unique species-specific responses to pathological stimuli, further complicating the use of these animal models to study oligodendrocytes. This was indeed shown to be the case in a comparative transcriptomic analysis of human and murine oligodendrocytes in Alzheimer’s disease research [60]. In fact, this study confirmed that the oligodendrocyte activation signature observed in human Alzheimer’s disease is largely distinct from those observed in mice. Possibilities to better understand the pathological mechanisms are, for example, the generation of novel animal modes based on identified molecular pathways that are critical for oligodendrocytes in PD, as well as the “humanization” of animal models through transplantation of human oligodendrocytes.

Human iPSC models have made significant progress, and their use, particularly for studying dopaminergic neurons, has increased significantly in the PD field [8,16]. However, obtaining iPSC-derived oligodendrocytes remains a challenge (see section “Why has the role of oligodendrocytes in PD pathogenesis remained so elusive?”). Although stem cell-derived models do not fully recapitulate the human brain in general, and a neurodegenerative disease due to several limitations in particular, they are highly valuable for studying the underlying molecular mechanisms of cells in isolation. Having robust oligodendrocyte models that can also be kept for an extended amount of time and expanding these to more complex systems, such as co-cultures and oligodendrocyte-harboring and myelinating 3D organoid models, will allow the examination of cell–cell communication and network building. These models should therefore—in parallel to advancing suitable in vivo models—be of highest priority.

Therefore, due to the limitations of all model systems, a concerted scientific approach combining the use of human imaging data and postmortem material, together with human in vitro iPSC platforms and rodent models, may provide some further insight to open scientific questions on this topic.

## Conclusions

Oligodendrocytes are a highly abundant cell type in the central nervous system and are essential for brain health and its physiological functions. Therefore, it is not surprising that recent transcriptomic studies have showcased that oligodendrocytes are linked with risk factors of PD and may be involved in the pathogenesis of the disease. Although these studies are extremely interesting and of great value, functional studies are needed to elucidate the role of oligodendrocytes and OPCs in this disease. Studying the causal role of oligodendrocytes, whether they are drivers or bystanders of pathology, the abundance and function of myelin on dopaminergic neurons, the response of oligodendrocytes to oxidative stress, and α-synuclein aggregation appears to be of utmost priority. The full picture requires an orchestrated effort combining the strengths of various in vivo and in vitro models, as well as the use of human brain tissue. Due to their high plasticity, even during adulthood, pharmaceutical targeting of oligodendrocytes in early disease stages could become a valuable therapeutic approach in PD research, with the aim of advancing their neuroprotective potential to slow, stop, or even prevent neurodegeneration in the first place.

## Abbreviations:

GWAS: genome-wide association studies
iPSC: induced pluripotent stem cell
MRI: magnetic resonance imaging
OPC: oligodendrocyte progenitor cell
PD: Parkinson’s disease
SNP: single-nucleotide polymorphism

## References

[1] Spillantini MG, Schmidt ML, Lee VM, Trojanowski JQ, Jakes R, Goedert M. Alpha-synuclein in Lewy bodies. Nature. 1997;388(6645):839–40. doi: 10.1038/42166 . 9278044

[2] Wong YC, Krainc D. α-synuclein toxicity in neurodegeneration: mechanism and therapeutic strategies. Nat Med. 2017;23(2):1–13. 10.1038/nm.4269 PMC8480197 28170377

[3] Kamath T, Abdulraouf A, Burris SJ, Langlieb J, Gazestani V, Nadaf NM, et al. Single-cell genomic profiling of human dopamine neurons identifies a population that selectively degenerates in Parkinson’s disease. Nat Neurosci. 2022;25(5):588–95. doi: 10.1038/s41593-022-01061-1 35513515 PMC9076534

[4] Okano H, Morimoto S. iPSC-based disease modeling and drug discovery in cardinal neurodegenerative disorders. Cell Stem Cell. 2022;29(2):189–208. doi: 10.1016/j.stem.2022.01.007 . 35120619

[5] Chia SJ, Tan E-K, Chao Y-X. Historical Perspective: Models of Parkinson’s Disease. Int J Mol Sci. 2020;21(7). doi: 10.3390/ijms21072464 ; PubMed Central PMCID: PMC7177377. 32252301 PMC7177377

[6] Burbulla LF, Song P, Mazzulli JR, Zampese E, Wong YC, Jeon S, et al. Dopamine oxidation mediates mitochondrial and lysosomal dysfunction in Parkinson’s disease. Science. 2017;357(6357):1255–61. doi: 10.1126/science.aam9080 ; PubMed Central PMCID: PMC6021018. 28882997 PMC6021018

[7] Uemura N, Uemura MT, Lo A, Bassil F, Zhang B, Luk KC, et al. Slow Progressive Accumulation of Oligodendroglial Alpha-Synuclein (α-Syn) Pathology in Synthetic α-Syn Fibril-Induced Mouse Models of Synucleinopathy. J Neuropathol Exp Neurol. 2019;78(10):877–90. doi: 10.1093/jnen/nlz070 ; PubMed Central PMCID: PMC6934438. 31504665 PMC6934438

[8] Kriks S, Shim J-W, Piao J, Ganat YM, Wakeman DR, Xie Z, et al. Dopamine neurons derived from human ES cells efficiently engraft in animal models of Parkinson’s disease. Nature. 2011;480(7378):547–51. doi: 10.1038/nature10648 ; PubMed Central PMCID: PMC3245796. 22056989 PMC3245796

[9] Dehestani M, Kessler W, Karmali N, Sun W, Volos P, Tsitkov S, et al. Single-cell transcriptomic changes in oligodendroglial lineage cells derived from Parkinson’s disease patient-iPSCs with LRRK2-G2019S mutation; 2024. 10.1016/j.isci.2026.116368 PMC13315888 42383004

[10] Simons M, Nave K-A. Oligodendrocytes: Myelination and Axonal Support. Cold Spring Harb Perspect Biol. 2015;8(1):a020479. doi: 10.1101/cshperspect.a020479 ; PubMed Central PMCID: PMC4691794. 26101081 PMC4691794

[11] Bryois J, Skene NG, Hansen TF, Kogelman LJA, Watson HJ, Liu Z, et al. Genetic identification of cell types underlying brain complex traits yields insights into the etiology of Parkinson’s disease. Nat Genet. 2020;52(5):482–93. doi: 10.1038/s41588-020-0610-9 32341526 PMC7930801

[12] Dean DC, Sojkova J, Hurley S, Kecskemeti S, Okonkwo O, Bendlin BB, et al. Alterations of Myelin Content in Parkinson’s Disease: A Cross-Sectional Neuroimaging Study. PLoS ONE. 2016;11(10):e0163774. doi: 10.1371/journal.pone.0163774 ; PubMed Central PMCID: PMC5051727. 27706215 PMC5051727

[13] Boshkovski T, Cohen-Adad J, Misic B, Arnulf I, Corvol J-C, Vidailhet M, et al. The Myelin-Weighted Connectome in Parkinson’s Disease. Mov Disord. 2022;37(4):724–33. doi: 10.1002/mds.28891 ; PubMed Central PMCID: PMC9303520. 34936123 PMC9303520

[14] Braak H, Del Tredici K. Poor and protracted myelination as a contributory factor to neurodegenerative disorders. Neurobiol Aging. 2004;25(1):19–23. doi: 10.1016/j.neurobiolaging.2003.04.001 . 14675725

[15] Wang Q, Wang M, Choi I, Sarrafha L, Liang M, Ho L, et al. Molecular profiling of human substantia nigra identifies diverse neuron types associated with vulnerability in Parkinson’s disease. Sci Adv. 2024;10(2):eadi8287. doi: 10.1126/sciadv.adi8287 ; PubMed Central PMCID: PMC10780895. 38198537 PMC10780895

[16] Kim TW, Piao J, Koo SY, Kriks S, Chung SY, Betel D, et al. Biphasic Activation of WNT Signaling Facilitates the Derivation of Midbrain Dopamine Neurons from hESCs for Translational Use. Cell Stem Cell. 2021;28(2):343–355.e5. doi: 10.1016/j.stem.2021.01.005 ; PubMed Central PMCID: PMC8006469. 33545081 PMC8006469

[17] Lee SW, Oh YM, Victor MB, Yang Y, Chen S, Strunilin I, et al. Longitudinal modeling of human neuronal aging reveals the contribution of the RCAN1–TFEB pathway to Huntington’s disease neurodegeneration. Nature Aging. 2024;4(1):95–109. doi: 10.1038/s43587-023-00538-3 38066314 PMC11456361

[18] Sun Z, Kwon J-S, Ren Y, Chen S, Walker CK, Lu X, et al. Modeling late-onset Alzheimer’s disease neuropathology via direct neuronal reprogramming. Science. 2024;385(6708):adl2992. doi: 10.1126/science.adl2992 . 39088624 PMC11787906

[19] Bergles DE, Richardson WD. Oligodendrocyte Development and Plasticity. Cold Spring Harb Perspect Biol. 2015;8(2):a020453. doi: 10.1101/cshperspect.a020453 ; PubMed Central PMCID: PMC4743079. 26492571 PMC4743079

[20] Livesey MR, Magnani D, Cleary EM, Vasistha NA, James OT, Selvaraj BT, et al. Maturation and electrophysiological properties of human pluripotent stem cell-derived oligodendrocytes. Stem Cells. 2016;34(4):1040–53. doi: 10.1002/stem.2273 ; PubMed Central PMCID: PMC4840312. 26763608 PMC4840312

[21] Wang S, Bates J, Li X, Schanz S, Chandler-Militello D, Levine C, et al. Human iPSC-derived oligodendrocyte progenitor cells can myelinate and rescue a mouse model of congenital hypomyelination. Cell Stem Cell. 2013;12(2):252–64. doi: 10.1016/j.stem.2012.12.002 ; PubMed Central PMCID: PMC3700553. 23395447 PMC3700553

[22] Ehrlich M, Mozafari S, Glatza M, Starost L, Velychko S, Hallmann A-L, et al. Rapid and efficient generation of oligodendrocytes from human induced pluripotent stem cells using transcription factors. Proc Natl Acad Sci U S A. 2017;114(11):E2243–E2252. doi: 10.1073/pnas.1614412114 ; PubMed Central PMCID: PMC5358375. 28246330 PMC5358375

[23] García-León JA, Kumar M, Boon R, Chau D, One J, Wolfs E, et al. SOX10 Single Transcription Factor-Based Fast and Efficient Generation of Oligodendrocytes from Human Pluripotent Stem Cells. Stem Cell Rep. 2018;10(2):655–72. doi: 10.1016/j.stemcr.2017.12.014 ; PubMed Central PMCID: PMC5830935. 29337119 PMC5830935

[24] James OG, Selvaraj BT, Magnani D, Burr K, Connick P, Barton SK, et al. iPSC-derived myelinoids to study myelin biology of humans. Dev Cell. 2021;56(9):1346–1358.e6. doi: 10.1016/j.devcel.2021.04.006 ; PubMed Central PMCID: PMC8098746. 33945785 PMC8098746

[25] Marton RM, Miura Y, Sloan SA, Li Q, Revah O, Levy RJ, et al. Differentiation and maturation of oligodendrocytes in human three-dimensional neural cultures. Nat Neurosci. 2019;22(3):484–91. doi: 10.1038/s41593-018-0316-9 ; PubMed Central PMCID: PMC6788758. 30692691 PMC6788758

[26] Orimo S, Uchihara T, Kanazawa T, Itoh Y, Wakabayashi K, Kakita A, et al. Unmyelinated axons are more vulnerable to degeneration than myelinated axons of the cardiac nerve in Parkinson’s disease. Neuropathol Appl Neurobiol. 2011;37(7):791–802. doi: 10.1111/j.1365-2990.2011.01194.x . 21696416

[27] Spaas J, van Veggel L, Schepers M, Tiane A, van Horssen J, Wilson DM, et al. Oxidative stress and impaired oligodendrocyte precursor cell differentiation in neurological disorders. Cell Mol Life Sci. 2021;78(10):4615–37. doi: 10.1007/s00018-021-03802-0 ; PubMed Central PMCID: PMC8195802. 33751149 PMC8195802

[28] Thorburne SK, Juurlink BH. Low glutathione and high iron govern the susceptibility of oligodendroglial precursors to oxidative stress. J Neurochem. 1996;67(3):1014–22. doi: 10.1046/j.1471-4159.1996.67031014.x . 8752107

[29] Arai T, Uéda K, Ikeda K, Akiyama H, Haga C, Kondo H, et al. Argyrophilic glial inclusions in the midbrain of patients with Parkinson’s disease and diffuse Lewy body disease are immunopositive for NACP/alpha-synuclein. Neurosci Lett. 1999;259(2):83–6. doi: 10.1016/s0304-3940(98)00890-8 . 10025563

[30] Wakabayashi K, Hayashi S, Yoshimoto M, Kudo H, Takahashi H. NACP/α-synuclein-positive filamentous inclusions in astrocytes and oligodendrocytes of Parkinson’s disease brains. Acta Neuropathol. 2000;99(1):14–20. 10651022 10.1007/pl00007400

[31] Macnair W, Calini D, Agirre E, Bryois J, Jäkel S, Kukanja P, et al. Single nuclei RNAseq stratifies multiple sclerosis patients into distinct white matter glial responses; 2022. 10.1016/j.neuron.2024.11.016 39708806

[32] Reyes JF, Rey NL, Bousset L, Melki R, Brundin P, Angot E. Alpha-synuclein transfers from neurons to oligodendrocytes. Glia. 2014;62(3):387–98. doi: 10.1002/glia.22611 . 24382629

[33] Wenning GK, Stefanova N, Jellinger KA, Poewe W, Schlossmacher MG. Multiple system atrophy: a primary oligodendrogliopathy. Ann Neurol. 2008;64(3):239–46. doi: 10.1002/ana.21465 . 18825660

[34] Adams L, Song MK, Yuen S, Tanaka Y, Kim Y-S. A single-nuclei paired multiomic analysis of the human midbrain reveals age- and Parkinson’s disease–associated glial changes. Nature Aging. 2024;4(3):364–78. doi: 10.1038/s43587-024-00583-6 38491288 PMC11361719

[35] Agarwal D, Sandor C, Volpato V, Caffrey TM, Monzón-Sandoval J, Bowden R, et al. A single-cell atlas of the human substantia nigra reveals cell-specific pathways associated with neurological disorders. Nat Commun. 2020;11(1):4183. doi: 10.1038/s41467-020-17876-0 32826893 PMC7442652

[36] Bae E-J, Pérez-Acuña D, Rhee KH, Lee S-J. Changes in oligodendroglial subpopulations in Parkinson’s disease. Mol Brain. 2023;16(1):65. doi: 10.1186/s13041-023-01055-5 37710343 PMC10500805

[37] Dehestani M, Kozareva V, Blauwendraat C, Fraenkel E, Gasser T, Bansal V. Transcriptomic changes in oligodendrocytes and precursor cells associate with clinical outcomes of Parkinson’s disease. Mol Brain. 2024;17(1):56. doi: 10.1186/s13041-024-01128-z 39138468 PMC11323592

[38] Martirosyan A, Ansari R, Pestana F, Hebestreit K, Gasparyan H, Aleksanyan R, et al. Unravelling cell type-specific responses to Parkinson’s Disease at single cell resolution. Mol Neurodegener. 2024;19(1):7. doi: 10.1186/s13024-023-00699-0 ; PubMed Central PMCID: PMC10799528. 38245794 PMC10799528

[39] Smajić S, Prada-Medina CA, Landoulsi Z, Ghelfi J, Delcambre S, Dietrich C, et al. Single-cell sequencing of human midbrain reveals glial activation and a Parkinson-specific neuronal state. Brain. 2022;145(3):964–78. doi: 10.1093/brain/awab446 ; PubMed Central PMCID: PMC9050543. 34919646 PMC9050543

[40] Walker CK, Roche JK, Sinha V, Roberts RC. Substantia nigra ultrastructural pathology in schizophrenia. Schizophr Res. 2018;197:209–18. doi: 10.1016/j.schres.2017.12.004 ; PubMed Central PMCID: PMC6013319. 29274737 PMC6013319

[41] Tomassy GS, Berger DR, Chen H-H, Kasthuri N, Hayworth KJ, Vercelli A, et al. Distinct profiles of myelin distribution along single axons of pyramidal neurons in the neocortex. Science. 2014;344(6181):319–24. doi: 10.1126/science.1249766 ; PubMed Central PMCID: PMC4122120. 24744380 PMC4122120

[42] Bacmeister CM, Huang R, Osso LA, Thornton MA, Conant L, Chavez AR, et al. Motor learning drives dynamic patterns of intermittent myelination on learning-activated axons. Nat Neurosci. 2022;25(10):1300–13. doi: 10.1038/s41593-022-01169-4 ; PubMed Central PMCID: PMC9651929. 36180791 PMC9651929

[43] Braak H, Del Tredici K, Gai WP, Braak E. Alpha-synuclein is not a requisite component of synaptic boutons in the adult human central nervous system. J Chem Neuroanat. 2000;20(3–4):245–52. doi: 10.1016/s0891-0618(00)00101-0 . 11207422

[44] Nieuwenhuys R. The greater limbic system, the emotional motor system and the brain. Prog Brain Res. 1996;107:551–80. doi: 10.1016/s0079-6123(08)61887-7 . 8782542

[45] Wei Z, Li X, Li X, Liu Q, Cheng Y. Oxidative Stress in Parkinson’s Disease: A Systematic Review and Meta-Analysis. Front Mol Neurosci. 2018;11:236. doi: 10.3389/fnmol.2018.00236 ; PubMed Central PMCID: PMC6041404. 30026688 PMC6041404

[46] Todorich B, Pasquini JM, Garcia CI, Paez PM, Connor JR. Oligodendrocytes and myelination: the role of iron. Glia. 2009;57(5):467–78. doi: 10.1002/glia.20784 . 18837051

[47] Mukherjee C, Kling T, Russo B, Miebach K, Kess E, Schifferer M, et al. Oligodendrocytes Provide Antioxidant Defense Function for Neurons by Secreting Ferritin Heavy Chain. Cell Metab. 2020;32(2):259–272.e10. doi: 10.1016/j.cmet.2020.05.019 ; PubMed Central PMCID: PMC7116799. 32531201 PMC7116799

[48] Giacci MK, Bartlett CA, Smith NM, Iyer KS, Toomey LM, Jiang H, et al. Oligodendroglia Are Particularly Vulnerable to Oxidative Damage after Neurotrauma In Vivo. J Neurosci. 2018;38(29):6491–504. doi: 10.1523/JNEUROSCI.1898-17.2018 ; PubMed Central PMCID: PMC6705954. 29915135 PMC6705954

[49] Mavroeidi P, Arvanitaki F, Karakitsou A-K, Vetsi M, Kloukina I, Zweckstetter M, et al. Endogenous oligodendroglial alpha-synuclein and TPPP/p25α orchestrate alpha-synuclein pathology in experimental multiple system atrophy models. Acta Neuropathol. 2019;138(3):415–41. 31011860 10.1007/s00401-019-02014-y PMC7289399

[50] Fang L-P, Bai X. Oligodendrocyte precursor cells: the multitaskers in the brain. Pflugers Arch. 2023;475(9):1035–44. doi: 10.1007/s00424-023-02837-5 ; PubMed Central PMCID: PMC10409806. 37401986 PMC10409806

[51] Birey F, Kloc M, Chavali M, Hussein I, Wilson M, Christoffel DJ, et al. Genetic and Stress-Induced Loss of NG2 Glia Triggers Emergence of Depressive-like Behaviors through Reduced Secretion of FGF2. Neuron. 2015;88(5):941–56. doi: 10.1016/j.neuron.2015.10.046 ; PubMed Central PMCID: PMC5354631. 26606998 PMC5354631

[52] Djogo T, Robins SC, Schneider S, Kryzskaya D, Liu X, Mingay A, et al. Adult NG2-Glia Are Required for Median Eminence-Mediated Leptin Sensing and Body Weight Control. Cell Metab. 2016;23(5):797–810. doi: 10.1016/j.cmet.2016.04.013 27166944

[53] Caldwell M, Ayo-Jibunoh V, Mendoza JC, Brimblecombe KR, Reynolds LM, Zhu Jiang XY, et al. Axo-glial interactions between midbrain dopamine neurons and oligodendrocyte lineage cells in the anterior corpus callosum. Brain Struct Funct. 2023;228(8):1993–2006. doi: 10.1007/s00429-023-02695-y ; PubMed Central PMCID: PMC10516790. 37668732 PMC10516790

[54] Auguste YSS, Ferro A, Kahng JA, Xavier AM, Dixon JR, Vrudhula U, et al. Oligodendrocyte precursor cells engulf synapses during circuit remodeling in mice. Nat Neurosci. 2022;25(10):1273–8. doi: 10.1038/s41593-022-01170-x ; PubMed Central PMCID: PMC9534756. 36171430 PMC9534756

[55] Holmes SE, Honhar P, Tinaz S, Naganawa M, Hilmer AT, Gallezot J-D, et al. Synaptic loss and its association with symptom severity in Parkinson’s disease. NPJ Parkinsons Dis. 2024;10(1):42. doi: 10.1038/s41531-024-00655-9 38402233 PMC10894197

[56] Zhang S-Z, Wang Q-Q, Yang Q-Q, Gu H-Y, Yin Y-Q, Li Y-D, et al. NG2 glia regulate brain innate immunity via TGF-β2/TGFBR2 axis. BMC Med. 2019;17(1):204. doi: 10.1186/s12916-019-1439-x ; PubMed Central PMCID: PMC6857135. 31727112 PMC6857135

[57] Falcão AM, van Bruggen D, Marques S, Meijer M, Jäkel S, Agirre E, et al. Disease-specific oligodendrocyte lineage cells arise in multiple sclerosis. Nat Med. 2018;24(12):1837–44. doi: 10.1038/s41591-018-0236-y ; PubMed Central PMCID: PMC6544508. 30420755 PMC6544508

[58] Kirby L, Jin J, Cardona JG, Smith MD, Martin KA, Wang J, et al. Oligodendrocyte precursor cells present antigen and are cytotoxic targets in inflammatory demyelination. Nat Commun. 2019;10(1):3887. doi: 10.1038/s41467-019-11638-3 ; PubMed Central PMCID: PMC6715717. 31467299 PMC6715717

[59] Jäkel S, Agirre E, Mendanha Falcão A, van Bruggen D, Lee KW, Knuesel I, et al. Altered human oligodendrocyte heterogeneity in multiple sclerosis. Nature. 2019;566(7745):543–7. doi: 10.1038/s41586-019-0903-2 ; PubMed Central PMCID: PMC6544546. 30747918 PMC6544546

[60] Pandey S, Shen K, Lee S-H, Shen Y-AA, Wang Y, Otero-García M, et al. Disease-associated oligodendrocyte responses across neurodegenerative diseases. Cell Rep. 2022;40(8):111189. doi: 10.1016/j.celrep.2022.111189 . 36001972

